# The First Neotropical Record of the Genus *Urodeta* (Lepidoptera: Elachistidae: Elachistinae) with Keys to the World Species and a Description of a New Species from Honduras

**DOI:** 10.3390/insects15120941

**Published:** 2024-11-28

**Authors:** Virginijus Sruoga

**Affiliations:** Institute of Biosciences, Life Sciences Center, Vilnius University, Saulėtekio Ave. 7, LT-10257 Vilnius, Lithuania; virginijus.sruoga@gmail.com

**Keywords:** microlepidoptera, mining moths, morphology, taxonomy, *Urodeta inerme* sp. nov., Central America

## Abstract

The genus *Urodeta* Stainton (Lepidoptera: Elachistidae: Elachistinae) is recorded for the first time from the Neotropical region, with the description of a new species, *Urodeta inerme* sp. nov., from Honduras. The new species is diagnosed and illustrated with photographs of the adults, genitalia of both sexes, and habitat. Identification keys to the world species of the genus *Urodeta*, based on male and female genitalia, are provided.

## Simple Summary

Moths of the genus *Urodeta* Stainton were studied, which are very small in comparison to many species of the subfamily Elachistinae Bruand (family Elachistidae Bruand). *Urodeta* is currently the smallest genus and, until 2009, was known only from the Mediterranean region. Over the past 17 years, its geographical range expanded considerably, reaching Australia, sub-Saharan Africa, and Asia. Here, we describe *Urodeta inerme* sp. nov. from the tropical dry forests of Honduras, representing the first record of *Urodeta* in Central America. This discovery not only enhances the Elachistinae diversity in the Neotropics but also largely extends the distribution range of *Urodeta*.

## 1. Introduction

*Urodeta* Stainton is a small genus of mining microlepidoptera belonging to the subfamily Elachistinae Bruand (Gelechioidea, Elachistidae). The moths are small to very small, with a wingspan ranging from 4 to 9.5 mm. However, about 50 percent of the currently known species have a wingspan of up to 6 mm. The labial palpi are usually very small, ranging from vestigial and barely visible to 0.6 times the length of the head width. The antennae are shorter than the forewing and rather broad, especially in males. The forewings usually have a dull pattern, grey or brownish, sometimes with inconspicuous spots. The hindwings are narrow or very narrow, with a length/width ratio reaching up to 14 in *U. trilobata* De Prins and Sruoga [1] or even 16 in *U. noreikai* Sruoga and De Prins [2]. The most remarkable characteristic of the genus, unique within the Elachistinae, is the anteriorly directed spines of the gnathos in the male genitalia, contrasting with other genera of the subfamily where the spines of the gnathos are directed posteriorly. Other distinctive characters in the male genitalia are the phallus distally fused with the ventral shield of the juxta and the absence of a digitate process. In the female genitalia, the apophyses anteriores extend from the middle of segment 8 and spread apart laterad, while in other Elachistinae species, the apophyses anteriores extend from the lateral sides of segment 8.

Nearly nothing is known about the life history of *Urodeta* species, except for the Mediterranean *U. hibernella* (Staudinger), which mines the leaves of *Cistus monspeliensis* L. [3], *Cistus ×ledon* Lam. and *C. ladanifer* L. [4], and *C. salviifolius* L. [5], and the Australian *Urodeta* sp., which mines the leaves of *Terminalia* sp. [6].

**Figure 1:**
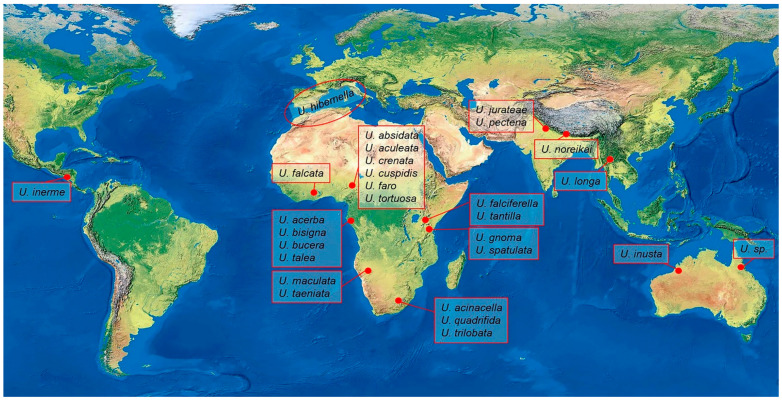
Global distribution of the genus *Urodeta*.

The extensive taxonomic history of the genus *Urodeta* was outlined by De Prins and Sruoga [1]. Since *Urodeta* was established by monotypy [3], only a single species, *U. hibernella*, from the Mediterranean region, has been known for about 140 years. However, the distribution range of *Urodeta* has been considerably expanded (Figure 1) by taxonomic studies over the last 17 years in Africa [1,7,8,9,10], Australia [6], and Asia [2,11,12].

A new species, *Urodeta inerme* sp. nov., is described from a series of specimens collected by Prof. J.R. Stonis in western Honduras in 2014, and the occurrence of the genus *Urodeta* is reported for the first time in the Neotropical region.

## 2. Materials and Methods

The study materials for this paper were obtained by Prof. Dr. Jonas R. Stonis, a senior researcher at the State Research Institute Nature Research Centre (Vilnius, Lithuania), who visited the Delegation of the European Union to Honduras and conducted voluntary research on the biological diversity of Honduran forests [13]. This initiative was part of two long-term programs between the European Union and Honduras: the Memorandum of Understanding between the Republic of Honduras and the European Union (“Forest Partnership”) and the Multiannual Indicative Program of the European Union for Honduras for 2021–2024, which includes Priority Area 1: “Sustainable Management of Natural Resources and Climate Change,” with the participation of the Honduran Institute of Forest Conservation, Protected Areas, and Wildlife (ICF) [13]. The moths were collected using a modern LepiLED lamp [14] and fluorescent lanterns powered by D dry cell batteries.

Adult specimens were examined externally using Nikon SMZ445 stereomicroscope (Nikon Corporation, Tokyo, Japan). The forewing length was measured along the costa from wing base to the apex of the terminal fringe scales with an ocular micrometer. The width of the head was measured between the inner edges of the antennal bases. Genitalia were prepared following the standard method described by Robinson [15], adapted for the Elachistinae as described by Traugott-Olsen and Nielsen [16]. The genitalia were studied, and some morphological structures were photographed in glycerol before permanent slide-mounting in Euparal. The male genital capsule was stained with fuchsin, the abdominal pelt, and the female genitalia with chlorazol black (Direct Black 38/Azo Black, Alfa Aesar GmbH & Co KG, Emmerich am Rhein, Germany). The genital morphology was examined using a Leica DM6 B microscope (Leica Microsystems, Wetzlar, Germany). The photographs of adults were taken using a Canon EOS 80D camera (Canon Inc., Tokyo, Japan) fitted with a MP-E 65 mm Canon macro lens, attached to a macro rail (Qool Rail, MJKZZ, China). Genitalia photographs were taken with a Leica DM6 B microscope and a Leica K3C digital camera. Zerene Stacker 1.0 with retouch function was used for image stacking. All images were optimized and grouped into plates using Adobe Photoshop CC 2019. The descriptive terminology of morphological structures follows Traugott-Olsen and Nielsen [16] and Kaila [6,17]. The material used in this paper will be deposited in the collection of the Museum für Naturkunde, Berlin, Germany, following publication.

## 3. Results

Key to the world species of *Urodeta* based on male genitalia (males of the following species are unknown and not included in the key: *U. acinacella*, *U. bisigna*, *U. falciferella*, *U. faro*, *U. longa*, *U. pectena*, *U. quadrifida,* and *U. tortuosa*).

1. Spinose knob of gnathos reduced ([18]: Figure 54) ....................................... *U. hibernella*

– Spinose knob of gnathos well developed, armed with spines directed cephalically ................................................................................................................................................. 2

2. Sacculus entirely separated from remaining valva as an elongate lobe ...................... 3

– Sacculus not separated from remaining valva ................................................................. 5

3. Valva divided into three distinct lobes (sacculus entirely separated, and termen of the remaining part of valva deeply emarginated, so appear divided into long and narrow lobes) ([1]: Figures 21–23) ................................................................................... *U. trilobata*

– Valva divided into two separate lobes (sacculus and the remaining part of the valva) ................................................................................................................................................. 4

4. Uncus well developed, as long as the width of the spinose knob of the gnathos; spinose knob of the gnathos large; vesica with different cornuti: one elongate plate-like, one near square plate-like, and numerous small spine-like cornuti ([9]: Figures 25–28) .................................................................................................................................... *U. acerba*

– Uncus not developed; spinose knob of the gnathos very small; vesica with several minute spines and two clusters of large cornuti (about 15–20 and 18–26), slightly different in size ([2]: Figures 5–11) .......................................................................... *U. noreikai*

5. Ventral margin of sacculus distinctly serrated ([9]: Figures 52–55) ................ *U. crenata*

– Ventral margin of sacculus not serrated ........................................................................... 6

6. Spinose knob of the gnathos divided into two separate lobes ([9]: Figures 39–41) ................................................................................................................................... *U. bucera*

– Spinose knob of gnathos not divided ............................................................................... 7

7. Inner processes of valvae fused apically and embedded with many small cusp-shaped spines ([9]: Figures 15–20) ...................................................................... *U. absidata*

– Valva without inner process embedded with spines ...................................................... 8

8. Phallus with a strongly sclerotized narrow band along the ventral margin ............... 9

– Phallus without a strongly sclerotized band along the ventral margin ...................... 12

9. Valvae are tightly fused together dorso–proximally; the spinose knob of the gnathos is rounded ([9]: Figures 74–76) ................................................................................. *U. talea*

– Valvae not fused together dorso–proximally; the spinose knob of the gnathos is elongated .................................................................................................................................... 10

10. Indentation of the distal margin of the juxta is wider than the width of the juxta lobe ([9]: Figures 35 and 36) ......................................................................................... *U. aculeata*

– Distal margin of the juxta is not indented ....................................................................... 11

11. Vesica with a cluster of minute internal spines and four large spine-shaped cornuti ([7]: Figures 33 and 34) ....................................................................................... *U. maculata*

– Vesica with a cluster of minute internal spines and two large claw-shaped cornuti ([10]: Figures 7–14) .................................................................................................. *U. falcata*

12. Vesica without cornuti ...................................................................................................... 13

– Vesica with cornuti ............................................................................................................ 14

13. Costa of the valva wrinkled, tapered to a sharp curved tip; cucullus short, about 1.5 times longer than wide ([6]: Figure 97) ....................... *U*. sp. reared from *Terminalia* sp.

– Costa of the valva smooth, without a sharp curved tip; cucullus long, about 10 times longer than wide (this paper, Figure 3) ................................................. *U. inerme* sp. nov.

14. Vesica with one cornutus .................................................................................................. 15

– Vesica with more than one cornutus ................................................................................ 16

15. Phallus about six–seven times longer than wide ([7]: Figures 35 and 36) ...... *U. taeniata*

– Phallus about three–four times longer than wide ([9]: Figures 58–63) ........... *U. cuspidis*

16. Phallus gradually tapered towards pointed apex.......................................................... 17

– Phallus apically not pointed ............................................................................................. 19

17. Cucullus narrow and long, about four times longer than broad; the spinose knob of the gnathos is oval ([8]: Figures 37–40) ................................................................. *U. gnoma*

– Cucullus short, about two times longer than broad; the spinose knob of the gnathos is rounded ............................................................................................................................ 18

18. Ventral shield of the juxta with the caudal margin mesially incised ([6]: Figures 95 and 96) ...................................................................................................................... *U. inusta*

– Ventral shield of the juxta with the caudal margin mesially not incised ([11]: Figures 9–13) ....................................................................................................................... *U. jurateae*

19. Ventral shield of the juxta with distinct apically rounded lobes; vesica with six large cornuti and numerous tiny spines ([8]: Figures 50 and 51) ............................... *U. tantilla*

– Ventral shield of the juxta without distinct lobes; vesica with two rows of large cornuti (4–5 in each row) and numerous tiny spines ([8]: Figures 44–47) ......... *U. spatulata*

Key to the world species of *Urodeta* based on female genitalia (females of the following species are unknown and not included in the key: *U. aculeata*, *U. crenata*, *U. cuspidis*, *U. falcata*, *U. gnoma*, *U. taeniata*, *U. tantilla*).

1. Corpus bursae with a signum ............................................................................................ 2

– Corpus bursae without a signum ..................................................................................... 14

2. Both pairs of apophyses (anteriores and posteriores) present ...................................... 3

– Apophyses anteriores absent ............................................................................................ 11

3. Apophyses posteriores well developed, with length not less than 0.7 of the length of abdominal segment 7 ........................................................................................................... 4

– Apophyses posteriores vestigial, not longer than 0.3 of length of abdominal segment 7 .............................................................................................................................................. 8

4. Ostium area covered by small spines ................................................................................ 5

– Ostium area without spines ................................................................................................ 6

5. Ductus bursae with longitudinal folds; the signum is sickle-shaped ([1]: Figures 6–10) ........................................................................................................................ *U. acinacella*

– Ductus bursae without longitudinal folds; the signum is formed by two weakly connected plates, each with a large spine and a few smaller ones ([1]: Figures 13–16) .............................................................................................................................. *U. quadrifida*

6. Ductus bursae coiled ........................................................................................................... 7

– Ductus bursae not coiled ([18]: Figure 55) ...................................................... *U. hibernella*

7. Signum formed by a weakly sclerotized plate with four large teeth in a row; ostium without sclerotized plates; antrum without internal spines ([6]: Figure 98) .... *U. inusta*

– Signum formed by one sickle-shaped spine and a weakly sclerotized transverse plate covered with tiny spines; ostium surrounded by longitudinal sclerotized plates; antrum with strong spines in the posterior part ([8]: Figures 41–43) .............. *U. falciferella*

8. Ductus bursae not coiled ([9]: Figures 42–49) ...................................................... *U. bucera*

– Ductus bursae coiled ........................................................................................................... 9

9. Antrum with weakly sclerotized longitudinal folds; the signum is rounded, dentate, and surrounded by spines arranged radially ([2]: Figures 12–17) .................. *U. noreikai*

– Antrum without sclerotized longitudinal folds; the signum is not rounded ............ 10

10. Signum formed from nine stout teeth, which slightly vary in size ([11]: Figures 18 and 19) ............................................................................................................................ *U. pectena*

– Signa consisting of two irregularly oval sclerotized plates, each with two or three short, stout spines (this paper, Figure 4) ............................................. *U. inerme* sp. nov.

11. Corpus bursae with two signa ([10]: Figures 17 and 18) ................................... *U. bisigna*

– Corpus bursae with one signum ...................................................................................... 12

12. Ductus bursae coiled ([9]: Figures 77–82) ............................................................... *U. talea*

– Ductus bursae not coiled ................................................................................................... 13

13. Corpus bursae with internal spines arranged in rows of 3–8; signum formed by an oval sclerotized plate with one large and several small spines ([1]: Figures 24–28) ................................................................................................................................ *U. trilobata*

– Corpus bursae without minute internal spines; signum formed from four stout teeth ([7]: Figures 30 and 31) ....................................................................................... *U. maculata*

14. Papillae anales with round, semitransparent spots where long setae arise; corpus bursae divided by a narrow prolonged constriction into two parts ([9]: Figures 29–32) .................................................................................................................................... *U. acerba*

– Papillae anales without round, semitransparent spots where long setae arise; corpus bursae not divided into two parts .................................................................................... 15

15. Corpus bursae narrow and long, about four times longer than wide ([9]: Figures 21 and 22) .................................................................................................................... *U. absidata*

– Corpus bursae rounded .................................................................................................... 16

16. Apophyses posteriores about 13 times longer than its width ([8]: Figures 48 and 49) ............................................................................................................................... *U. spatulata*

– Apophyses posteriores absent or not longer than seven times its width ............................................................................................................................................... 17

17. Posterior margin of sternum 8 strongly sclerotized and folded, forming a wide pocket ([9]: Figures 85–88) .............................................................................................. *U. tortuosa*

– Posterior margin of sternum 8 not folded ...................................................................... 18

18. Apophyses anteriores short, about 3–6 time longer than wide ................................... 19

– Apophyses anteriores absent ([6]: Figure 97) ............. *U*. sp. reared from *Terminalia* sp.

19. Antrum large, oval, and strongly sclerotized, with about 24 large and several small internal spines ([12]: Figures 3–6) ........................................................................... *U. longa*

– Antrum without internal spines ....................................................................................... 20

20. Colliculum membranous, with dense internal spines; dorsal wall with a large, strongly sclerotized paired plate ([11]: Figures 14 and 15) .............................. *U. jurateae*

– Colliculum strongly sclerotized, with minute internal spines; dorsal wall without a strongly sclerotized paired plate ([9]: Figures 66–71) ............................................. *U. faro*

**Figure 2:**
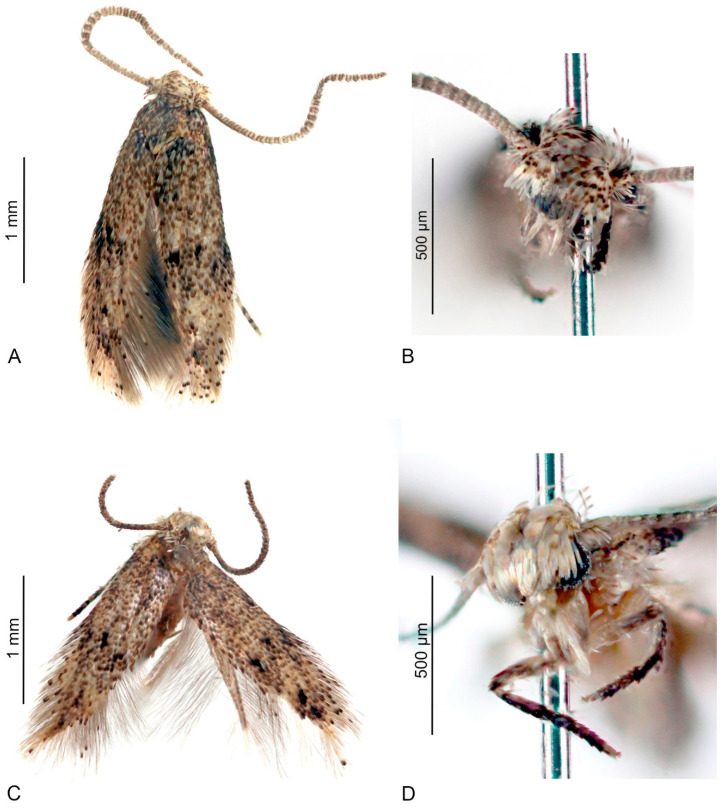
*Urodeta inerme* sp. nov.: (**A**) male, holotype; (**B**) same, head, frontal view; (**C**) female, paratype; (**D**) same, head, frontal view.

**Figure 3:**
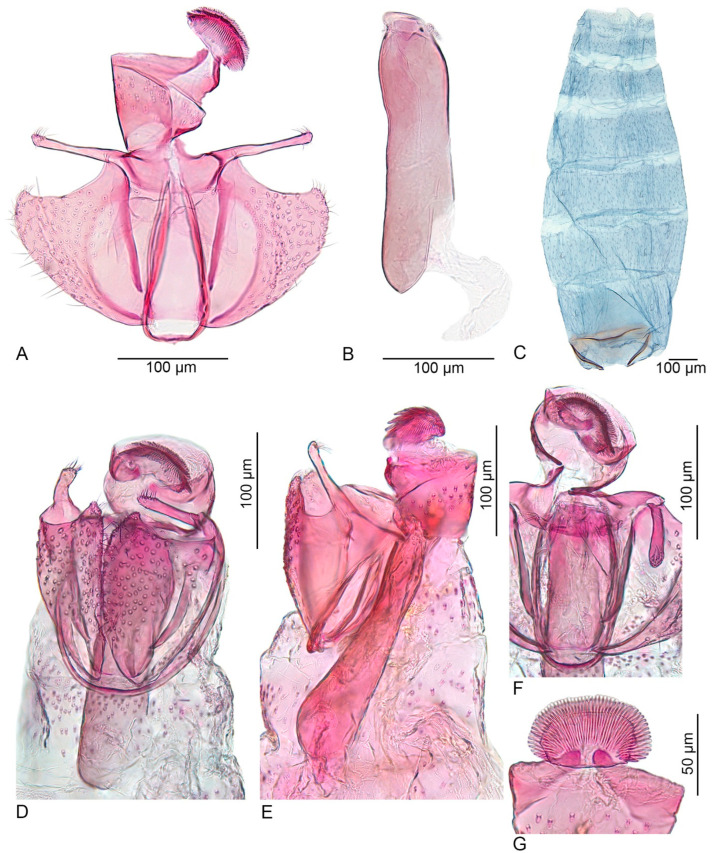
*Urodeta inerme* sp. nov.: (**A**) male genitalia (phallus removed), holotype; (**B**) phallus, holotype; (**C**) male abdominal pelt, holotype; (**D**) male genitalia, ventro–lateral view, paratype; (**E**) male genitalia, lateral view, paratype; (**F**) central part of male genitalia, paratype; (**G**) spinose knob of gnathos, ventro–apical view, paratype; (**A**–**C**) genitalia prep. VS493; (**D**,**F**) genitalia prep. VS495; (**E**,**G**) genitalia prep. VS492; (**D**–**G**) in glycerol before permanent mounting in Euparal.

**Figure 4:**
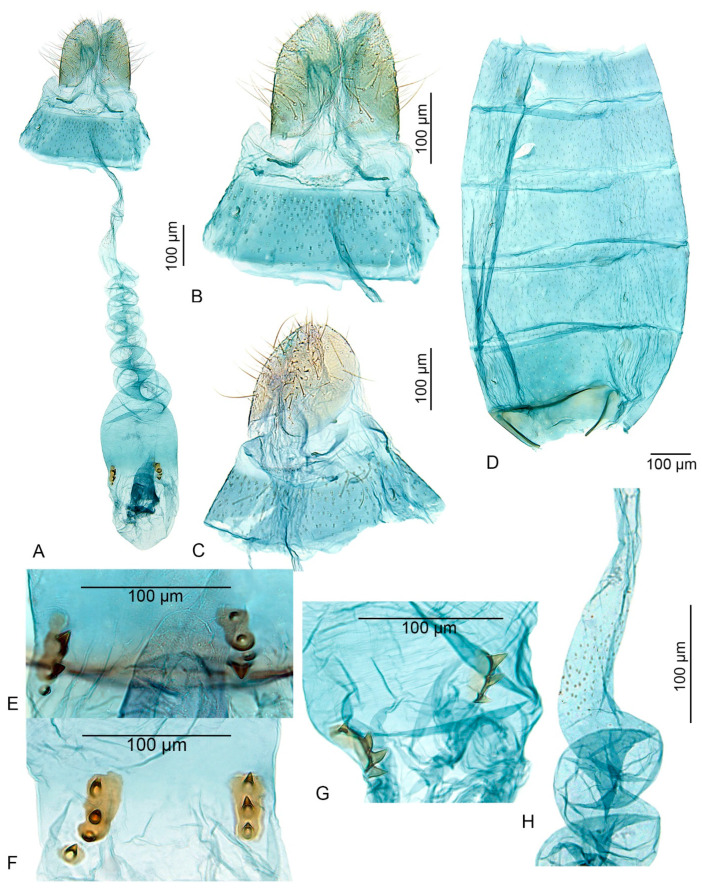
*Urodeta inerme* sp. nov., paratypes: (**A**) female genitalia, general view; (**B**) distal part of female genitalia, ventral view; (**C**) distal part of female genitalia, ventro–lateral view; (**D**) female abdominal pelt; (**E**,**F**) signa, ventral view; (**G**) signa, lateral view; (**H**) distal part of ductus bursae; (**A**,**B**,**E**) genitalia prep. VS491; (**C**,**D**,**G**) genitalia prep. VS494; (**F**,**H**) genitalia prep. VS488; (**E**) in glycerol before permanent mounting in Euparal.

**Figure 5:**
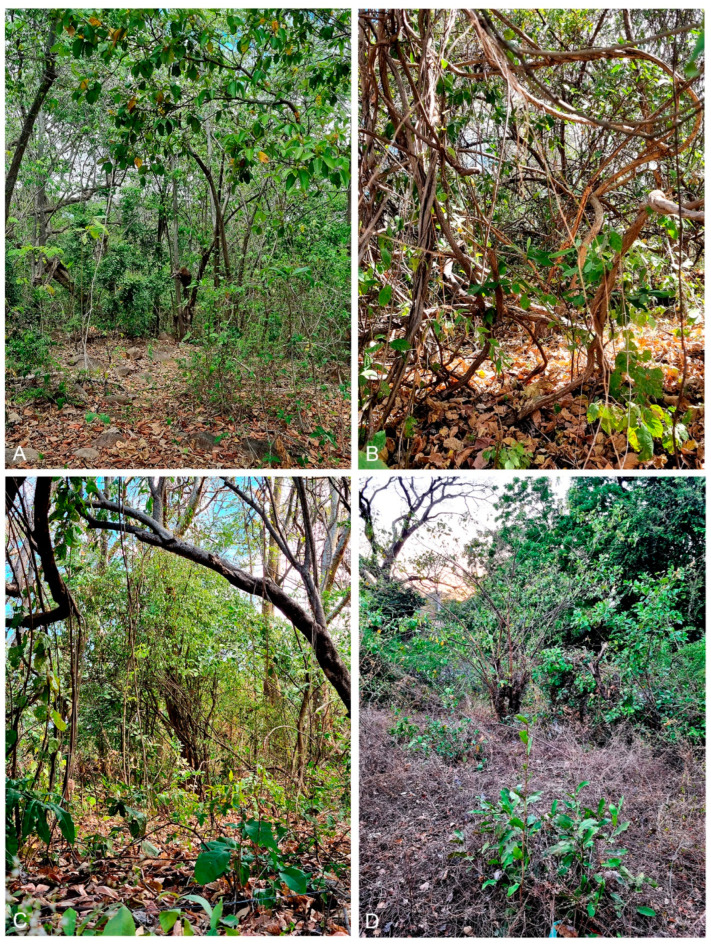
Sampling habitats of *Urodeta inerme* sp. nov. in the tropical dry forests in Honduras: (**A**) Isla Zacate Grande, Coyalito (Las Piletas); (**B**,**C**) Isla del Tigre, Amapala, Playa Grande; (**D**) San Lorenzo env. Photographs courtesy of Jonas R. Stonis.

*Urodeta inerme* sp. nov.

(Figure 1, Figure 2, Figure 3, Figure 4 and Figure 5)

urn:lsid:zoobank.org:act:05C855DC-041A-4474-9769-3AF6DD9C1222Material examined: Holotype: ♂, Honduras, Isla Zacate Grande, El Moray (Restaurante Terra Mar), 10 m, 13°21′33″ N, 87°35′57″ W, 18–19.ii.2023, leg. J.R. Stonis. Genitalia slide VS493. Paratypes: 1♂, Honduras, San Lorenzo, 1.5 km E by Pan American Hwy, left side, approx. 40 m, 13°25′59″ N, 87°25′24″ W, 6–7.ii.2023, leg. J.R. Stonis. Genitalia slide VS492; 1♀, Honduras, the Pacific, Isla del Tigre, Amapala, Playa Grande, approx. 20 m, 13°16′26″ N, 87°39′39″ W, 8.ii.2023, leg. J.R. Stonis. Genitalia slide VS488; 1♂, 1♀, Honduras, the Pacific, Isla Zacate Grande, El Moray (Restaurante Terra Mar), 20 m, 13°21′28″ N, 87°36′6″ W, 15–16.ii.2023, leg. J.R. Stonis. Genitalia slides VS489, VS494; 2♂, Honduras, the Pacific, Isla Zacate Grande, El Moray (Restaurante Terra Mar), 20 m, 13°21′30″ N, 87°36′5″ W, 20.iii.2023, leg. J.R. Stonis. Genitalia slide VS495; 1♂, 1♀, Honduras, the Pacific, Isla Zacate Grande, Coyalito (Las Piletas), 50 m, at light, 13°18′45″ N, 87°36′59″ W, 23–24.iii.2023, leg. J.R. Stonis. Genitalia slide VS491.

Diagnosis: *Urodeta inerme* is a small, pale-colored species with indistinct wing markings. In wing pattern and male genitalia, the new species is comparable with *U. crenata* Sruoga and De Prins, known from northern Cameroon (for external characters and male genitalia refer to [9]: Figures 50–55). The main differences in male genitalia between *U. inerme* and *U. crenata* are (1) the ventral margin of the sacculus is smooth in *U. inerme*, whereas it is serrated in *U. crenata*; (2) the spinose knob of the gnathos is large and oval in *U. inerme*, whereas it is small and rounded in *U. crenata*; (3) the apex of the phallus is blunt in *U. inerme*, whereas it is long and pointed in *U. crenata*. The female genitalia of *U. inerme* are highly distinctive, with signa consisting of a pair of longitudinal plates with a few short and stout spines, a coiled ductus bursae, and vestigial apophyses posteriores. As such, *U. inerme* cannot be confused with any other known species of *Urodeta*.

Male (Figure 2A,B): Forewing length 1.9–2.3 mm; wingspan 4.2–5.0 mm (*n* = 5). Head: frons shiny, beige–white; vertex and neck tuft beige–white, mottled with dark brown tips of scales; labial palpus straight, very short, about 0.5 times the width of the head, beige–white; scape beige–white, mottled with dark brown; pecten beige–white; flagellum grey–brown, weakly annulated with paler rings. Thorax, tegula, and forewing strongly mottled with scales, basally greyish white and distally dark brown; two irregular blackish brown spots transversally arranged just before the middle of the wing; fringe scales beige–white with some blackish brown-tipped scales. Hindwing beige–grey, its fringe scales somewhat paler.

Female (Figure 2C,D): the forewing length is 1.8–2.2 mm, and the wingspan is 4.0–4.8 mm (*n* = 3). It is similar to the male, but the flagellum is slightly thinner.

Male genitalia (Figure 3A–G): The uncus is short, about as long as the width of the spinose knob of the gnathos. The spinose knob of the gnathos is large, oval-shaped, 1.4 times as long as wide, and oriented posteriorly. The valva is short and broad; the ventral margin of the sacculus is convex, distally strongly concave, forming a sharp angle when meeting the cucullus; the cucullus is long and narrow, about 10 times longer than wide, ventrally directed. The ventral shield of the juxta is strongly sclerotized, 2.5 times as long as wide, basally broad, and apically slightly tapered; the lateral membranous extension of the juxta is apically bilobed and partly surrounds the phallus. The vinculum is U-shaped and narrow. The phallus is apically fused to the juxta, longer than the valva, slightly dilated in the proximal part, and the apex is truncated; the insertion of the ductus ejaculatorius dorso is laterally directed; the caecum is small; vesica without cornuti. Abdominal segments 7 and 8 are simple, without modifications.

Female genitalia (Figure 4A–H): The papilla analis is short and setose. Apophyses posteriores vestigial, visible only as tiny extensions basolaterally. The tergum 8 is very short, not sclerotized; apophyses anteriores are weakly sclerotized except at the apices, extending from the central part of the segment and spreading apart laterad. The ostium bursae is situated in the membrane between sterna 7 and 8, with a ventral margin with some broad and short spines. The antrum membranous is funnel-shaped, as long as the length of the apophysis anterioris. The colliculum is tube-shaped, weakly sclerotized, and 1.6 times the length of the antrum. The ductus bursae is long, spirally coiled, and posteriorly has several minute internal spines. The corpus bursae is oval-shaped, with a pair of signa consisting of two irregularly oval sclerotized plates, each provided with two or three short, stout spines. Additionally, there are one or two small, separated sclerotized plates with a single short, stout spine that may be situated near the anterior end of the signum. The corpus bursae is without internal spines.

Biology: All currently available specimens were collected at a light trap from February to March, during the peak of the dry season. Otherwise, the biology of this species remains unknown.

Flight period: based on the specimens available, adults fly in February and March.

Distribution: so far, this species occurs in the tropical dry forests along the Pacific coast in south–western Honduras (Figure 5A–D).

Etymology: from the Latin *inermis*, meaning “unarmed”, in reference to the absence of cornuti.

## 4. Discussion

The Elachistinae remain scarcely studied in the Neotropical region, especially in Central America, where only one species, *Stephensia armata* Sruoga, has been described from Belize [19]. Despite the sampling efforts conducted in various regions of the world, no species of *Urodeta* has been recorded from the Neotropics. The discovery of *Urodeta inerme* sp. nov. from Honduras provides the first record of the genus in Central America.

The new species is assigned to the genus *Urodeta* based on key apomorphies in the structure of the genitalia. In males, these include the anteriorly directed spines of the gnathos, the phallus distally fused with the ventral shield of the juxta, and the absence of a digitate process. In the female genitalia, the apophyses anteriores extend from the middle of segment 8 and spread apart laterad, which undoubtedly indicates an association with this genus.

Based on the geographical distance between the records from other continents, it suggests that the *Urodeta* might be much more diverse and widespread in the Americas and worldwide. However, additional sampling is needed, not only to determine the real species diversity of Neotropical *Urodeta* but also to delimit the geographic distribution of the already known species. Unfortunately, the immature stages and host plants of *Urodeta inerme* sp. nov. are still unknown, as are the vast majority of species in the genus.

Currently, the genus *Urodeta* comprises 27 species worldwide (Figure 1), including a new species described in this paper and one described but not named species from Australia [6]. So far, the highest diversity of *Urodeta* species (approximately 71%) is known from the Afrotropical region. This is likely due to the relatively extensive sampling conducted in that region by J. and W. De Prins [1,8,9,10], W. Mey, and K. Ebert [7]. In other biogeographical regions, the number of known species is significantly lower: four species are reported from the Oriental region, two from the Australian region, one from the extreme western edge of the Palearctic region (Mediterranean), and now, one from the Neotropical region (Figure 1).

As collection attempts correlate with the number of currently recognized species, it can be assumed that only after conducting adequate surveys can we identify the real centers of species diversity of *Urodeta*. Considering that the species of the genus are now found in tropical or near-tropical areas in almost all continents, the greatest diversity might actually be somewhere else, e.g., in Australia, Asia, or Central and South America.

## Data Availability

All data are contained within the article.
